# Two Sympatric *Spodoptera* Species Could Mutually Recognize Sex Pheromone Components for Behavioral Isolation

**DOI:** 10.3389/fphys.2019.01256

**Published:** 2019-09-27

**Authors:** Qi Yan, Xiao-Long Liu, Yu-Lei Wang, Xiao-Qin Tang, Zhi-Jie Shen, Shuang-Lin Dong, Jian-Yu Deng

**Affiliations:** ^1^Key Laboratory of Integrated Management of Crop Disease and Pests, Ministry of Education, Department of Entomology, Nanjing Agricultural University, Nanjing, China; ^2^Department of Plant Protection, Zhejiang A& F University, Hangzhou, China; ^3^College of Plant Sciences, Tibet Agricultural and Animal Husbandry University, Nyingchi, China

**Keywords:** *Spodoptera*, sympatric species, sex pheromone, reproductive isolation, multispecies management, mating disruption

## Abstract

*Spodoptera exigua* and *S. litura* are two sympatric species in China and many other countries. Both moths employ a multiple component sex pheromone blend, including a common component Z9,E12-14:OAc, and two specific components Z9-14:OH and Z11-16:OAc for *S. exigua*, and one specific component Z9,E11-14:OAc for *S. litura.* For the two species, it has been well documented that males are able to recognize and behaviorally attracted by their species-specific sex pheromone, which functions as a means of reproductive isolation, but whether males could mutually recognize pheromone components of its sympatric species is unknown. In the present study, the electroantennogram (EAG) and field evaluation were conducted to address this topic. The EAG recordings revealed that males of each species could significantly respond to specific components of its sympatric species, although the response values were lower than that to its own major component. In field tests, the specific components Z9-14:OH and Z11-16:OAc of *S. exigua* strongly inhibited the male catches of *S. litura* to its conspecific sex pheromone, while specific component Z9,E11-14:OAc of *S. litura* significantly reduced the male catches of *S. exigua* to its sex pheromone. Furthermore, the combined lure of the two species completely inhibited male catches of *S. litura*, and significantly decreased the male catches of *S. exigua*, compared to the species-specific lure alone. The results demonstrated that males of the two sibling species could perceive the specific components of its counterpart, suggesting that mutual recognition of pheromone components may function to strengthen the behavioral isolation between the two species. Our study has added new knowledge to the reproductive isolation via sex pheromone communication system in sympatric moth species, and provided a base for designing of mating disruption tactics targeting multispecies by using insect sex pheromones.

## Introduction

Lepidoptera is the second largest insect group, including about 150,000 species in the world. Therein, it is common that two and more species share the same geographical location and occurrence time. For these sympatric species, the species-specific sex pheromones in addition to morphological and physiological characteristics, play an important role in the reproductive isolation (Roelofs et al., 2002; Allison and Cardé, 2016). The Lepidoptera sex pheromones have been identified from virgin female moths of more than 660 species, and almost all insects have a multicomponent pheromone system (Ando and Yamakawa, 2011; Ando, 2018). The sex pheromones in different species are mainly diverse in chemical structure and composition of the pheromone components. In addition, some relative species that share same components, the differences in ratio of the components could be largely responsible for reproductive isolation, such as the case of *Helicoverpa armigera* and *H. assulta*. These two sibling species share the same two sex pheromone components of Z9-16:Ald and Z11-16:Ald, but in opposite ratios (Piccardi et al., 1977; Nesbitt et al., 1980; Kehat and Dunkelblum, 1990; Sugie et al., 1991; Li et al., 2017).

It worth noting that, in hundreds of identified pheromone components in Lepidoptera, many components are shared by several and even tens of different species (Ando, 2018). Of these species that are sympatric, the species specific components may contribute to the reproductive isolation (Linn et al., 1986; Saveer et al., 2014). Some field trapping studies have suggested that one species can recognize sex pheromone component of other sympatric species. A specific pheromone component of *H. virescens*, Z9-14:Ald, played an intraspecific role in sexual communication, and also served as behavioral antagonist for *H. zea* to avoid the cross-attraction (Haile et al., 1973; Roach, 1975; Carpenter et al., 1984; Hansson et al., 1986; Grant et al., 1987; Christensen et al., 1990). As a minor component of the sex pheromone in *Chilo suppressalis*, Z11-16:OH did not increase the attractiveness of the pheromone for conspecific males in the field, but did increase the sex pheromone specificity (Chen et al., 2018). Similarly, Z3,Z13-18:OAc of *Synanthedon scoliaeformis* is a behavioral antagonist against *S. tipuliformis* males, and E3,Z13-18:OAc of *S. tipuliformis* is an antagonist against *S. scoliaeformis* males (Mozūraitis and Būda, 2013).

The two *Spodoptera* species, the beet armyworm (*S. exigua* Hübner) and the tobacco cutworm (*S. litura* Fabricius), are both polyphagous agricultural pests worldwide. They share a similar geographic distribution and broadly occurrence time in China and many other countries. Furthermore, the two species have a common sex pheromone component Z9,E12-14:OAc, while each species also uses some specific components. In *S. exigua*, several pheromone components, Z9,E12-14:OAc, Z9-14:OH, Z11-16:OAc, Z9-14:OAc, and Z9,E12-14:OH, were identified successively in the female pheromone glands of different populations (Persoons et al., 1981; Tumlinson et al., 1981, 1990; Mochizuki et al., 1993; Mitchell and Tumlinson, 1994; Dong and Du, 2002), with the former three components at the ratio of 7:3:1 attracting the most number of males in east Asia (Jung et al., 2003; Zhao et al., 2018). Different from *S. exigua*, the female *S. litura* showed highest attraction for males with Z9,E11-14:OAc and Z9,E12-14:OAc in a ratio of 9:1 (Tamaki et al., 1973). It has been well documented that males of the two species are able to recognize and behaviorally attracted by their species-specific sex pheromones without cross-attraction, and therefore, we hypothesize that males of these two species may mutually recognize the species-specific components of its sympatric counterpart, and thus to strengthen the behavioral isolation between the two sibling species.

To verify the above hypothesis, we in the present study, measured the mutual recognition of sex pheromone components between *S. litura* and *S. exigua* on both physiological and behavioral levels, providing evidence that males of the two *Spodoptera* species can recognize the species-specific components of its sympatric counterpart.

## Materials and Methods

### Insects

Larvae of *Spodoptera litura* and *S. exigua* were collected in flowering cabbage fields of Nanjing (Jiangsu province, China; 32.01°N, 118.63°E). The larvae were fed on an artificial diet in the laboratory in 14L:10D at 26 ± 1°C and 65 ± 5% relative humidity (Huang et al., 2002). Pupae were separated by sex and placed in separate cages. After the pupae emerged, the moths were fed with 10% honey solution.

### Chemicals

The pheromone components (*Z*,*E*)-9,12-tetradecadienyl acetate (Z9,E12-14:OAc), (*Z*)-9-tetradecenyl acetate (Z9-14:OAc), (*Z*)-11-hexadecenyl acetate (Z11-16:OAc), and (*Z*,*E*)-9,11-tetradecadienyl acetate (Z9,E11-14:OAc) were purchased from Nimord Inc. (Changzhou, China). The purity of each chemical was >98% checked by Gas Chromatography.

### Electroantennogram (EAG) Recordings

Solutions of the four pheromone components (Z9,E12-14:OAc, Z9-14:OAc, Z11-16:OAc, and Z9,E11-14:OAc) were prepared in distilled hexane at different concentrations (0.01, 0.1, 1.0, 10, and 100 ng/μl). Antennae of virgin males (2 days after eclosion) were cut off at the base, and the terminal distal segment was excised. The antenna was connected by conductive gel (Spectra 360, Fairfield, NJ, United States) to two electrodes. A filter paper strip (2.5 cm × 1.0 cm) containing 10 μl of a test solution was inserted into a Pasteur pipette. The thin end of Pasteur pipette was placed through a hole in a metal tube, which directed a clean airstream over the antenna. By using an air flow controller (CS-55, Syntech Inc., Netherlands), each test compound was expelled into the airstream by a puff of air (4 ml/sec) through the Pasteur pipette. Compounds were tested from low to high dosage, and ach dosage was tested at least five antenna, with a 10 s interval for recovery of the antenna. The antennal signal was amplified tenfold, and converted to a digital signal by DC amplifier interface (IDAC, Syntech Inc., Netherlands). The signals were recorded with EAGPro software (version 2.0, Syntech Inc., Netherlands).

### Field Trapping Tests

All field trappings were carried out in asparagus fields of Pinghu (Zhejiang, China; 30.65°N, 121.02°E) between August and September in 2017 and 2018. The synthetic pheromone compounds, Z9,E12-14:OAc, Z9-14:OAc, Z11-16:OAc, and Z9,E11-14:OAc were dissolved in distilled hexane (20 mg/ml). Then related solutions were added to rubber septa (black rubber, 12 mm O.D., Pherobio Inc., Beijing, China) that were used as dispensers. Control septa were loaded with hexane (50 μl) only. After all the hexane was volatilized, each group of rubber septa was packed with seal bag (7 cm × 4 cm) and stored at 4°C until use for field trapping. As a lure, every rubber septa was placed at the center of sticky trap (30 cm × 30 cm bottom plate with a roof, Nimord Inc., Changzhou, China), and were hung separately 1.0 m above ground level at intervals of 10 m. Four replicates for each treatment were tested, and the captured males were counted every week.

### Statistical Analysis

Data obtained in each field test were analyzed by one-way ANOVA, and pair wise comparisons among traps were performed with Tukey-Kramer Test with *p*-values adjusted for multiple comparisons. To homogenize the variance, means were transformed using log(x + 0.5) transformation. All statistical analyses were performed with R version 3.3.2 (R Development Core Team, 2016).

## Results

### EAG Responses of Males

**FIGURE 1:**
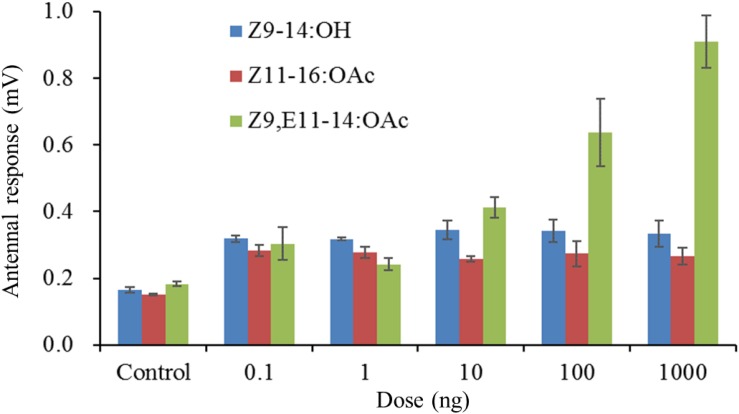
Electroantennogramphic (EAG) responses (mean mV ± SE, n = 6) of antennae of male *Spodoptera litura* to synthetic pheromone components. Z9-14:OH and Z11-16:OAc are the pheromone components of *S. exigua*, and Z9,E11-14:OAc is the pheromone component of *S. litura*. Response values for each component at all test doses are all significantly higher than that for the control at *P* < 0.05 by Tukey-Kramer Test.

**FIGURE 2:**
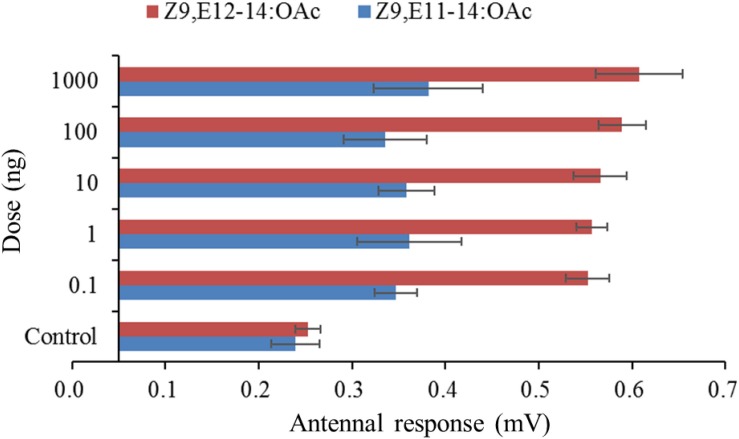
Electroantennographic (EAG) responses (mean mV ± SE, n = 6) of antennae of male *Spodoptera exigua* to synthetic pheromone components. Z9,E12-14:OAc is the pheromone component of *S. exigua*, and Z9,E11-14:OAc is the pheromone component of *S. litura*. Response values for each component at all test doses are all significantly higher than that for the control at *P* < 0.05 by Tukey-Kramer Test.

In order to make sure whether the males of *S. exigua* or *S. litura* could response to the specific pheromone component of its sympatric species, the species-specific components were singly tested. The male antennae responses of *S. litura* to synthetic components were shown in Figure 1. In comparison to the control (hexane), two *S. exigua* specific pheromone components (Z9-14:OH and Z11-16:OAc) elicited significantly higher EAG activity to the males. However, at the higher doses of 100 and 1000 ng, the responses stimulated by Z9-14:OH and Z11-16:OAc were weaker than Z9,E11-14:OAc, the major pheromone component of *S. litura*. Similarly for the male antennae of *S. exigua*, the *S. litura* specific pheromone component Z9,E11-14:OAc elicited significantly higher EAG responses at all tested doses, but these EAG values were much lower than those by Z9,E12-14:OAc, the major pheromone component of *S. exigua* (Figure 2).

### Field Trapping Tests

**FIGURE 3:**
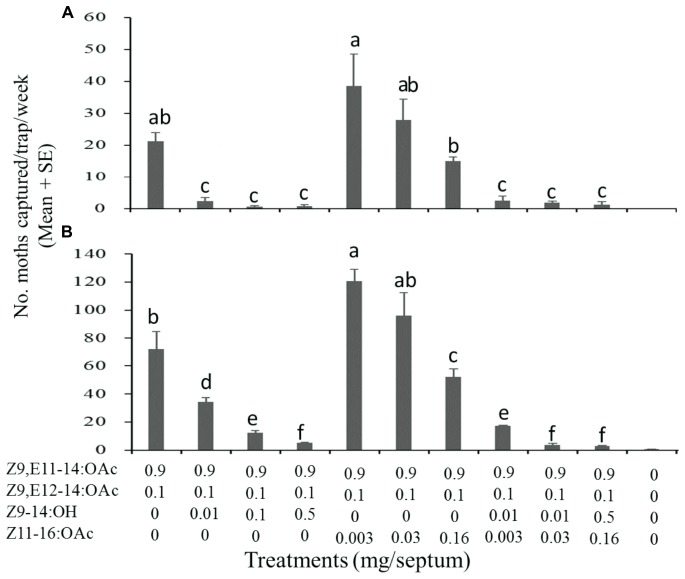
Catches of *Spodoptera litura* males in asparagus fields in Pinghu city of Zhejiang Province (China) in traps baited with synthetic pheromone components. **(A)** Test on 16 August-12 September 2017, 4 replicates; **(B)** Test on 19 August-17 September 2018, 4 replicates. Values within each test followed by different letter are significantly different at *P* < 0.01 by Tukey-Kramer Test.

Firstly, it was evaluated regarding the effect of two *S. exigua* specific components on the attractiveness of *S. litura* sex pheromone to the conspecific males (Figure 3). The ratio of Z9-14:OH and Z11-16:OAc used in the test was same as that in the pheromone blend of *S. exigua*. The results were generally consistent over the two test years. Compared to the binary mixture of Z9,E11-14:OAc and Z9,E12-14:OAc (9:1, the sex pheromone of *S. litura*), the addition of Z9-14:OH into the mixture significantly decreased number of males captured in the trap baited with the pheromone mixture. For the second specific component Z11-16:OAc, inhibition effect was also observed at the high usage of 0.016 mg/septa, while the effect was not significant at the lower usage of 0.003 and 0.03 mg/septa. An exemption was the use usage of 0.003 mg/septa in the 2018 test, where addition of Z11-16:OAc to the binary mixture increased the male catches (Figure 3B). When both Z9-14:OH and Z11-16:OAc mixed with the sex pheromone of *S. litura*, this inhibition effect was more significant, showing that the numbers of captured males were significantly lower at all doses.

**FIGURE 4:**
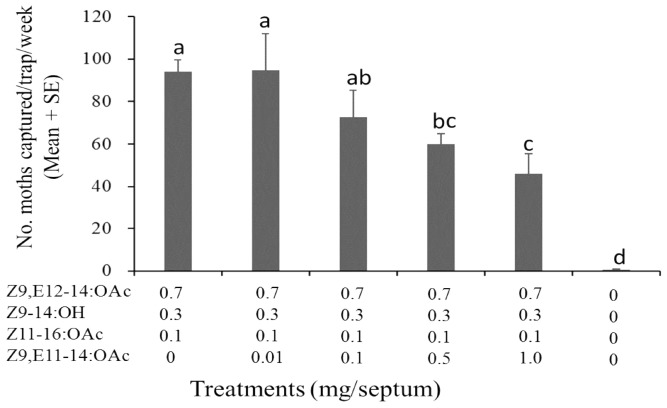
Catches of *Spodoptera exigua* males in asparagus fields in Pinghu city of Zhejiang Province (China) in traps baited with synthetic pheromone components (19 August–17 September 2018, 4 replicates). Values followed by a different letter are significantly different at *P* < 0.05 by Tukey-Kramer Test.

Meanwhile, the effect of Z9,E11-14:OAc (the specific component of *S. litura*) on the sex pheromone of *S. exigua* was also examined. It showed that the addition of Z9,E11-14:OAc to the ternary blends of Z9,E12-14:OAc, Z9-14:OH, and Z11-16:OAc (7:3:1) had no effects at the low dose, but as the ratio of Z9,E11-14:OAc increased to 0.5, the number of captured males was decreased significantly (Figure 4).

Then, the combined lure that includes the sex pheromones of *S. litura* and *S. exigua* was evaluated for the attractiveness to males of both species in 2 years (Table 1). In comparison to the lure containing *S. litura* sex pheromones alone, almost no *S. litura* male was attracted in the combined lures. Compared to the lure containing *S. exigua* sex pheromones alone, the number of male catches of *S. exigua* by the combined lures was significantly decreased in 2017.

**TABLE 1:** Catches of *Spodoptera litura* and *Spodoptera exigua* males in asparagus fields in Zhejiang Province (China) in traps baited synthetic pheromone components.

	**Lure component (mg/rubber septum)*^a^***				**Captured males/trap/week*^b^***	
		
	**Z9,E11-14:OAc**	**Z9,E12-14:OAc**	**Z9-14:OH**	**Z11-16:OAc**	***S. litura***	***S. exigua***
Test 1^c^	0.9	0.1	0	0	21.4 ± 5.2	1.0 ± 0.7 c
	0.9	0.8	0.3	0.1	0	14.0 ± 3.7 b
	0	0.7	0.3	0.1	0	27.3 ± 8.6 a
	0	0	0	0	0	0
Test 2	0.9	0.1	0	0	57.3 ± 20.7 a	0.3 ± 0.2 b
	0.9	0.8	0.3	0.1	0.8 ± 0.4 b	64.9 ± 15.8 a
	0	0.7	0.3	0.1	0.4 ± 0.3 b	77.8 ± 7.3 a
	0	0	0	0	0	0.2 ± 0.2 b

**^*a*^*The mixture of Z9,E11-14:OAc and Z9,E12-14:OAc (9:1) is the sex pheromone of *S. litura*, and the other mixture of Z9,E12-14:OAc, Z9-14:OH and Z11-16:OAc (7:3:1) is the sex pheromone of *S. exigua*. The combined lure is the mixture of the two sex pheromones. *^*b*^*Mean ± SE, values within each test followed by a different letter are significantly different at *P* < 0.05 by Tukey-Kramer Test. ^*c*^Test 1: 16 August-12 September 2017, 4 replicates; Test 2: 19 August-17 September 2018, 4 replicates.*

## Discussion

Our study demonstrated conclusively that males of *S. exigua* and *S. litura* could mutually perceive the specific pheromone components of its sympatric counterpart. The EAG recordings revealed that males can respond not only to conspecific sex pheromone components, but also to interspecific components. In the field trapping tests, the specific components of *S. exigua*, Z9-14:OH and Z11-16:OAc, significant inhibits the attraction of the sex pheromone of *S. litura*. Similarly, the component of *S. litura*, Z9,E11-14:OAc, inhibits the attraction of the sex pheromone of *S. exigua*. The results confirm the hypothesis that *S. exigua* and *S. litura* can mutually recognize the interspecific component(s) from the conspecific sex pheromone.

Pheromone signals is an essential component of mate recognition in sexual communication of moths. In some sympatric moth species, filed trapping experiments have shown that specific pheromone components of one species may serve as antagonists for other sympatric species to avoid the cross-attraction (Christensen et al., 1990; Mozūraitis and Būda, 2013; Chen et al., 2018). Our field tests also have shown that there is no cross-attraction between *S. exigua* and *S. litura*, when the sex pheromones were employed to attract conspecific males respectively in South China (unpublished data), suggesting that the species specific components play roles in mate recognition. The present study further confirmed at both electro-physiologically and behaviorally that these two sibling species could mutually perceive the specific pheromone components of its sympatric counterpart. Therefore, it is a common phenomenon that sympatric species can distinguish the interspecific pheromone components as behavioral antagonist, in order to avoid the cross-attraction. An interesting example was the sibling species *S. littoralis* and *S. litura*, which share four pheromone compounds in pheromone glands, while *S. littoralis* still has seven more compounds; however, *S. litura* males did not discriminate between conspecific and heterospecific calling females (Saveer et al., 2014), suggesting that *S. litura* males could not recognize the specific pheromone components of *S. littoralis*.

The recognition of interspecific pheromone components is supported by recent molecular studies on the sex pheromone perception. Pheromone binding proteins (PBPs) and pheromone receptors (PRs) are two important olfactory protein classes in the recognition of sex pheromone components (Prestwich, 1996; Krieger et al., 2004; Kaissling, 2007; Leal, 2007, 2013). Our previous research on ligand binding affinity of PBPs are consistent with the present results of EAG tests (Table 2). Both SlitPBP1 and SlitPBP2 of *S. litura* have high affinities with Z9-14:OH, and SlitPBP1 showed high affinity with Z11-16:OAc (Liu et al., 2012, 2013). Similarly, SexiPBP1 and SexiPBP2 of *S. exigua* have a high affinity with Z9,E11-14:OAc (Liu et al., 2015). Furthermore, studies have shown that some PRs did not tune to their own sex pheromones components but to the components of other species. SlitOR16, a PR of *S. litura*, did not respond to its pheromone component Z9,E11-14:OAc, but responded strongly to Z9-14:OH, a pheromone component of *S. exigua* (Zhang et al., 2015). Coincidentally, a *Sesamia inferens* PR, SinfOR27 did not respond to all three pheromone components, but reacted strongly to Z9,E12-14:OAc, a common pheromone component of other noctuid species (Zhang et al., 2014). Taken together, it can be suggested that, over the long history of evolution, the sympatric species could not only perceive conspecifics sex pheromone components, but also may monitor the components of other insects, so as to ensure the species specificity of sex pheromone communication and thus the reproductive isolation at the premating stage.

**TABLE 2:** Dissociation constants (*Ki* values) of sex pheromone compounds to pheromone binding proteins (PBPs) of *Spodoptera litura* and *Spodoptera exigua* (Summarized from Liu et al., 2012, 2013, 2015).

**Pheromone components**	**Dissociation constants (*Ki* value, μM)*^a^***					
	
	**SlitPBP1**	**SlitPBP2**	**SlitPBP3**	**SexiPBP1**	**SexiPBP2**	**SexiPBP3**
Z9,E11-14:OAc	1.07	3.24	12.39	1.15	3.24	–*^*b*^*
Z9,E12-14:OAc	0.57	2.20	4.77	0.81	2.99	–
Z9-14:OH	2.01	1.36	3.56	0.46	1.24	2.25
Z11-16:OAc	3.52	–	–	1.05	–	–

*^*a*^Dissociation constants (*Ki* value:) of each competitor were calculated from the corresponding IC50 values (the concentration of competitor halving the initial fluorescence intensity), using the following equation: Ki = [IC_50_]/(1 + [1-NPN]/K_1__–NPN_), where [1-NPN] is the free concentration of 1-NPN and K_1__–NPN_ is the dissociation constant of the complex protein/1-NPN. The lower of the *Ki* value, the better of the binding affinity between ligand components and PBP. ^*b*^The Ki values are represented as “–” for ligands with IC_50_ undetected or unable to be calculated.*

Over the past few decades, the different techniques of insect sex pheromone were used for control of insect pests including *Spodoptera spp.*, in which the mating disruption has been the most successful technique (Cardé and Minks, 1995; Witzgall et al., 2010; Guerrero et al., 2014). However, the utilization of this technique has rarely targeted multiple species simultaneously. Compared to the species-specific sex pheromones of *S. exigua* or *S. litura* alone, the combined lure, which mixed two species’ sex pheromones, completely attracted none males of *S. litura*, and the male catches of *S. exigua* significantly decreased. This provides us an attractive possibility that mating disruption can be used to target two and more sympatric species by carefully select pheromone component(s) that are recognized by those insect species.

## Conclusion

The males of *S. litura* and *S. exigua* could perceive pheromone components of its relative counterpart and reduce behavioral response to its own pheromone blend when mixed with component(s) of its relative counterpart. Our present study provides an evidence that two sympatric moth species can mutually recognize the interspecific sex pheromones. This mutual recognition would be of great significance for the reproductive isolation, especially for the phylogenetically closed and sympatric species that are more likely share one or more sex pheromone components and employ behavioral isolation as a key mechanism in the reproductive isolation.

## Data Availability Statement

All datasets generated for this study are included in the manuscript/supplementary files.

## Author Contributions

QY and S-LD conceived and designed the experiments and wrote the manuscript. X-LL and X-QT performed the electroantennogram experiments. J-YD, Y-LW, and Z-JS arranged the field tests and preliminary data analysis. QY, X-LL, and S-LD worked on data analysis. All authors reviewed the final manuscript.

## Conflict of Interest

The authors declare that the research was conducted in the absence of any commercial or financial relationships that could be construed as a potential conflict of interest.
